# Birth and Infant Outcomes Following Laboratory-Confirmed SARS-CoV-2 Infection in Pregnancy — SET-NET, 16 Jurisdictions, March 29–October 14, 2020

**DOI:** 10.15585/mmwr.mm6944e2

**Published:** 2020-11-06

**Authors:** Kate R. Woodworth, Emily O’Malley Olsen, Varsha Neelam, Elizabeth L. Lewis, Romeo R. Galang, Titilope Oduyebo, Kathryn Aveni, Mahsa M. Yazdy, Elizabeth Harvey, Nicole D. Longcore, Jerusha Barton, Chris Fussman, Samantha Siebman, Mamie Lush, Paul H. Patrick, Umme-Aiman Halai, Miguel Valencia-Prado, Lauren Orkis, Similoluwa Sowunmi, Levi Schlosser, Salma Khuwaja, Jennifer S. Read, Aron J. Hall, Dana Meaney-Delman, Sascha R. Ellington, Suzanne M. Gilboa, Van T. Tong, CDC COVID-19 Response Pregnancy and Infant Linked Outcomes Team, COVID-19 Pregnancy and Infant Linked Outcomes Team (PILOT), Augustina Delaney, Jason Hsia, Kellianne King, Mirna Perez, Megan Reynolds, Aspen Riser, Maria Rivera, Christina Sancken, John Sims, Ashley Smoots, Margaret Snead, Penelope Strid, Tineka Yowe-Conley, Laura Zambrano, Lauren Zapata, Susan Manning, Veronica Burkel, Amanda Akosa, Carolyne Bennett, Isabel Griffin, John Nahabedian, Suzanne Newton, Nicole M. Roth, Neha Shinde, Erin Whitehouse, Daniel Chang, Charise Fox, Yousra Mohamoud, Florence Whitehill

**Affiliations:** ^1^CDC COVID-19 Response; ^2^New Jersey Department of Health; ^3^Massachusetts Department of Public Health; ^4^Tennessee Department of Health; ^5^New York State Department of Health; ^6^Georgia Department of Public Health; ^7^Michigan Department of Health and Human Services; ^8^Minnesota Department of Health; ^9^Nebraska Department of Health and Human Services; ^10^Oklahoma State Health Department; ^11^Los Angeles County Department of Public Health; ^12^Puerto Rico Department of Health; ^13^Pennsylvania Department of Health; ^14^California Department of Public Health; ^15^North Dakota Department of Health; ^16^Houston Health Department; ^17^Vermont Department of Health.; CDC; CDC; CDC; CDC; CDC; CDC; CDC; CDC; CDC; CDC; CDC; CDC; CDC; CDC; CDC; Massachusetts Department of Public Health; Eagle Medical Services; Eagle Global Scientific; Eagle Global Scientific; Eagle Global Scientific; Eagle Global Scientific; Eagle Global Scientific; Eagle Global Scientific; Eagle Global Scientific; Epidemic Intelligence Service; Oak Ridge Institute for Science and Education; Oak Ridge Institute for Science and Education; Oak Ridge Institute for Science and Education; Oak Ridge Institute for Science and Education.

*On November 2, 2020, this report was posted online as an *MMWR* Early Release.*

Pregnant women with coronavirus disease 2019 (COVID-19) are at increased risk for severe illness and might be at risk for preterm birth (*1*–*3*). The full impact of infection with SARS-CoV-2, the virus that causes COVID-19, in pregnancy is unknown. Public health jurisdictions report information, including pregnancy status, on confirmed and probable COVID-19 cases to CDC through the National Notifiable Diseases Surveillance System.* Through the Surveillance for Emerging Threats to Mothers and Babies Network (SET-NET), 16 jurisdictions collected supplementary information on pregnancy and infant outcomes among 5,252 women with laboratory-confirmed SARS-CoV-2 infection reported during March 29–October 14, 2020. Among 3,912 live births with known gestational age, 12.9% were preterm (<37 weeks), higher than the reported 10.2% among the general U.S. population in 2019 (*4*). Among 610 infants (21.3%) with reported SARS-CoV-2 test results, perinatal infection was infrequent (2.6%) and occurred primarily among infants whose mother had SARS-CoV-2 infection identified within 1 week of delivery. Because the majority of pregnant women with COVID-19 reported thus far experienced infection in the third trimester, ongoing surveillance is needed to assess effects of infections in early pregnancy, as well the longer-term outcomes of exposed infants. These findings can inform neonatal testing recommendations, clinical practice, and public health action and can be used by health care providers to counsel pregnant women on the risks of SARS-CoV-2 infection, including preterm births. Pregnant women and their household members should follow recommended infection prevention measures, including wearing a mask, social distancing, and frequent handwashing when going out or interacting with others or if there is a person within the household who has had exposure to COVID-19.^†^

SET-NET conducts longitudinal surveillance of pregnant women and their infants to understand the effects of emerging and reemerging threats.^§^ Supplementary pregnancy-related information is reported for women with SARS-CoV-2 infection (based on detection of SARS-CoV-2 in a clinical specimen by molecular amplification detection testing^¶^) during pregnancy through the day of delivery. As of October 14, 2020, 16 jurisdictions** have contributed data. Pregnancy status was ascertained through routine COVID-19 case surveillance or through matching of reported cases with other sources (e.g., vital records, administrative data) to identify or confirm pregnancy status. Data were abstracted using standard forms^††^; sources include routine public health investigations, vital records, laboratory reports, and medical records. Chi-squared tests were performed to test for statistically significant (p<0.05) differences in proportion of outcomes between women reported to have symptomatic infection and those reported to have asymptomatic infection using SAS (version 9.4; SAS Institute). This activity was reviewed by CDC and was conducted consistent with applicable federal law and CDC policy.^§§^

Jurisdictions reported 5,252 pregnant women with SARS-CoV-2 infection. Among these women, 309 (5.9%) were presumed to have ongoing pregnancies (no outcome reported and not past their estimated due date plus 90 days for reporting lag), and 501 (9.5%) did not have pregnancy outcomes reported and were either missing an estimated due date or presumed lost to follow-up. This report focuses on the 4,442 women with known pregnancy outcomes (84.6% of 5,252 women).

The median age of women was 28.9 years, and 46.0% were Hispanic or Latina (Hispanic) ethnicity (Table 1). At least one underlying medical condition was reported for 1,564 (45.1%) women, with prepregnancy obesity (body mass index ≥30 kg/m^2^) (35.1%) being the most commonly reported. Most (84.4%) women had infection identified in the third trimester (based on date of first positive test result or symptom onset). Symptom status was known for 2,691 (60.6%) women, 376 (14.0%) of whom were reported to be asymptomatic.

**TABLE 1:** Demographics, underlying medical conditions, and SARS-CoV-2 infection characteristics of pregnant women with known pregnancy outcomes, by symptom status — SET-NET, 16 jurisdictions, March 29–October 14, 2020

Characteristic	No. of women (%) [Total no. of women with available information]			
	Total	With symptomatic* infection	With asymptomatic infection	Unknown symptom status
	N = 4,442 (100.0)	N = 2,315 (52.1)	N = 376 (8.5)	N = 1,751 (39.4)
**Age group, yrs**	**[3,097]**	**[1,883]**	**[298]**	**[916]**
Median (IQR)	28.9 (24.4–34.0)	30.0 (24.7–34.0)	28.0 (24.2–33.7)	30.0 (24.2–34.0)
<20	167 (5.4)	97 (5.2)	26 (8.7)	44 (4.8)
20–24	654 (21.1)	390 (20.7)	63 (21.1)	201 (21.9)
25–29	735 (23.7)	454 (24.1)	74 (24.8)	207 (22.6)
30–34	870 (28.1)	530 (28.1)	75 (25.2)	265 (28.9)
35–39	525 (17.0)	326 (17.3)	46 (15.4)	153 (16.7)
≥40	146 (4.7)	86 (4.6)	14 (4.7)	46 (5.0)
**Race/Ethnicity**	**[3,523]**	**[2,026]**	**[308]**	**[1,189]**
Hispanic or Latina	1,622 (46.0)	876 (43.2)	138 (44.8)	608 (51.1)
Asian, non-Hispanic	122 (3.5)	78 (3.8)	5 (1.6)	39 (3.3)
Black, non-Hispanic	741 (21.0)	410 (20.2)	80 (26.0)	251 (21.1)
White, non-Hispanic	914 (25.9)	592 (29.2)	78 (25.3)	244 (20.5)
Multiple or other race, non-Hispanic	124 (3.5)	70 (3.5)	7 (2.3)	47 (4.0)
**Health insurance^†^**	**[2,697]**	**[1,363]**	**[289]**	**[1,045]**
Private	1,074 (39.8)	613 (45.0)	124 (42.9)	337 (32.2)
Medicaid	1,442 (53.5)	645 (47.3)	146 (50.5)	651 (62.3)
Other	80 (3.0)	39 (2.9)	10 (3.5)	31 (3.0)
Self-pay/None	101 (3.7)	66 (4.8)	9 (3.1)	26 (2.5)
**Underlying medical conditions**	**[3,471]**	**[1,998]**	**[322]**	**[1,151]**
Any underlying condition^§^	1,564 (45.1)	902 (45.1)	135 (41.9)	527 (45.8)
Cardiovascular disease	35 (1.0)	31 (1.6)	3 (0.9)	1 (0.1)
Chronic hypertension	55 (1.6)	30 (1.5)	10 (3.1)	15 (1.3)
Chronic lung disease	100 (2.9)	85 (4.3)	10 (3.1)	5 (0.4)
Diabetes mellitus^¶^	74 (2.1)	56 (2.8)	7 (2.2)	11 (1.0)
Immunosuppression	23 (0.7)	16 (0.8)	4 (1.2)	3 (0.3)
Obesity (BMI ≥30 kg/m^2^)	1,217 (35.1)	684 (34.2)	97 (30.1)	436 (37.9)
Other**	26 (0.7)	22 (1.1)	3 (0.9)	1 (0.1)
**Pregnancy related complications^††^**	**[2,794]**	**[1,673]**	**[270]**	**[851]**
Pregnancy induced hypertension	211 (7.6)	124 (7.4)	24 (8.9)	63 (7.4)
Gestational diabetes mellitus	228 (8.2)	141 (8.4)	21 (7.8)	66 (7.8)
**Trimester of SARS-CoV-2 infection^§§^**	**[3,309]**	**[2,031]**	**[295]**	**[983]**
First trimester (<14 wks)	13 (0.4)	11 (0.5)	1 (0.3)	1 (0.1)
Second trimester (14–27 wks)	502 (15.2)	347 (17.1)	24 (8.1)	131 (13.3)
Third trimester (≥28 wks)	2,794 (84.4)	1,673 (82.4)	270 (91.5)	851 (86.6)

**Abbreviations:** BMI = body mass index; COVID-19 = coronavirus disease 2019.

*Inclusive of women reported as symptomatic on the COVID-19 case report form (https://www.cdc.gov/coronavirus/2019-ncov/php/reporting-pui.html) or who had any symptoms reported on the COVID-19 case report form regardless of completion of the symptom status variable.

^†^ Latest known insurance during pregnancy or at delivery.

^§^ Includes all listed for all women, and gestational diabetes mellitus and pregnancy induced hypertension for women with infection identified in the third trimester. Pregnancy itself is not considered an underlying condition.

^¶^ Includes either type 1 or type 2 diabetes, does not include gestational diabetes.

** Other conditions include neurologic conditions or disabilities, chronic renal disease, chronic liver disease, psychiatric disorders, and autoimmune disorders.

^††^ Among women with SARS-CoV-2 infection in third trimester.

^§§^ Calculated as either date of specimen collection for first positive test, or symptom onset if exact date of specimen collection was missing.

Among 4,527 fetuses and infants, the outcomes comprised 4,495 (99.3%) live births (including 79 sets of twins and one set of triplets), 12 (0.3%) pregnancy losses at <20 weeks’ gestation, and 20 (0.4%) losses at ≥20 weeks’ gestation (Table 2). Among 3,912 infants with reported gestational age, 506 (12.9%) were preterm, including 149 (3.8%) at <34 weeks and 357 (9.1%) at 34–37 weeks. Frequency of preterm birth did not differ by maternal symptom status (p = 0.62), including among women hospitalized at the time of infection (p = 0.81, Fisher’s exact test). Among 3,486 (77.6%) live births with weight, gestational age, and sex reported, 198 (5.7%) were small for gestational age.^¶¶^ Twenty-eight (0.6%) infants were reported to have any birth defect; among 23 infants for whom timing of maternal SARS-CoV-2 infection during pregnancy was known, 17 (74%) were born to mothers with infection identified in the third trimester. Nine (0.2%) in-hospital neonatal deaths were reported. Among term infants (≥37 weeks’ gestation), 9.3% were admitted to an intensive care unit (ICU); however, reason for admission was often missing.

**TABLE 2:** Pregnancy and birth outcomes among pregnant women with laboratory-confirmed SARS-CoV-2 infection by symptom status* — SET-NET, 16 jurisdictions, March 29–October 14, 2020

Characteristic	No. of outcomes (%) [Total no. of women with available information]			
	Total	Women with symptomatic infection^†^	Women with asymptomatic infection	Women with no symptom status reported
	N = 4,442	N = 2,315 (52.1)	N = 376 (8.5)	N = 1,751 (39.4)
**Days from first positive RT-PCR test to pregnancy outcome**	**[3,278]**	**[2,104]**	**[278]**	**[894]**
Median (IQR)	17.5 (1–58)	23 (3–61)	1 (0–12)	12 (1–58)
**Induction of labor**	**[3,846]**	**[2,113]**	**[264]**	**[1,469]**
Induced	1,091 (28.4)	593 (28.1)	78 (29.5)	420 (28.6)
**Delivery type**	**[3,920]**	**[2,145]**	**[285]**	**[1,490]**
Vaginal	2,589 (66.0)	1,403 (65.4)	195 (68.4)	991 (66.5)
Cesarean	1,331 (34.0)	742 (34.6)	90 (31.6)	499 (33.5)
Emergent	110 (39.6)	72 (42.6)	11 (37.9)	27 (33.8)
Non-emergent	168 (60.4)	97 (57.4)	18 (62.1)	53 (66.3)
**Pregnancy outcome**	**[4,527] ^§^**	**[2,372]**	**[384]**	**[1,771]**
Live birth	4,495 (99.3)	2,355 (99.3)	379 (98.7)	1,761 (99.4)
Pregnancy loss	32 (0.7)	17 (0.7)	5 (1.3)	10 (0.6)
Pregnancy loss <20 weeks	12 (0.3)	10 (0.4)	2 (0.5)	0 (0.0)
Pregnancy loss ≥20 weeks	20 (0.4)	7 (0.3)	3 (0.8)	10 (0.6)
**Gestational age among live births**	**[3,912]**	**[2,137]**	**[287]**	**[1,488]**
Term (≥37 weeks)	3,406 (87.1)	1,840 (86.1)	244 (85.0)	1,322 (88.8)
Preterm (<37 weeks)	506 (12.9)	297 (13.9)	43 (15.0)	166 (11.2)
Late preterm (34–36 weeks)	357 (9.1)	211 (9.9)	28 (9.8)	118 (7.9)
Moderate preterm (32–33 weeks)	50 (1.3)	32 (1.5)	6 (2.1)	12 (0.8)
Very preterm (28–31 weeks)	69 (1.8)	41 (1.9)	6 (2.1)	22 (1.5)
Extremely preterm (<28 weeks)	30 (0.8)	13 (0.6)	3 (1.0)	14 (0.9)
**Infant ICU admission among term live births,^¶^ n/N (%)**	279/2,995 (9.3)	158/1,558 (10.1)	15/173 (8.7)	106/1,264 (8.4)
Birth defects among live births,** n/N (%)	28/4,447 (0.6)	18/2,330 (0.8)	2/371 (0.5)	8/1,746 (0.5)

**Abbreviations:** ICU = intensive care unit; IQR = Interquartile range; RT-PCR = reverse transcription–polymerase chain reaction.

* Chi-squared tests of association was performed to compare outcomes between women with symptomatic and asymptomatic infection for induction of delivery, cesarean delivery, pregnancy loss, preterm birth, infant ICU admission, and birth defects and was found to be statistically nonsignificant (p>0.1) for all.

^†^ Inclusive of women reported as symptomatic on the COVID-19 case report form (https://www.cdc.gov/coronavirus/2019-ncov/php/reporting-pui.html) or who had any symptoms reported on the COVID-19 case report form regardless of completion of the symptom status variable.

^§^ Pregnancy outcomes include 79 sets of twins and one set of triplets; therefore, number exceeds the number of women.

^¶^ Among term (≥37 weeks) infants only, reason for admission could include need for isolation of an otherwise asymptomatic infant based on possible SARS-CoV-2 exposure.

** Includes congenital heart defects (seven), cleft lip and/or palate (four), chromosomal abnormalities (four), genitourinary (four), gastrointestinal (two), cerebral cysts (one), talipes equinovarus (one), developmental dysplasia of the hip (one), supernumerary digits (one) and five had no birth defects specified. Total exceeds 28 because some infants had multiple birth defects reported.

Information on infant SARS-CoV-2 testing was reported from 13 jurisdictions; among 923 infants with information, 313 (33.9%) were not tested. Among 610 (21.3%) infants for whom molecular test results were reported, 16 (2.6%) results were positive (Table 3), including 14 for whom the timing of the mothers’ infection during pregnancy was reported. The percent positivity was 4.3% (14 of 328) among infants born to women with documentation of infection identified ≤14 days before delivery and 0% (0 of 84) among those born to women with documentation of infection identified >14 days before delivery.

**TABLE 3:** Characteristics of laboratory-confirmed infection among infants born to pregnant women with laboratory-confirmed SARS-CoV-2 infection — SET-NET, 13* jurisdictions, March 29–October 14, 2020

Characteristic	No. of infants (%) [Total no. of infants with available information]			
	Total	Not tested or missing data^†^	RT-PCR positive results	RT-PCR negative results
	N = 2,869 (100.0)	N = 2,259 (78.7)	N = 16 (0.6)^§^	N = 594 (20.7)
**Maternal symptom status**	**[1,871]**	**[1,475]**	**[13]**	**[383]**
Asymptomatic	231 (12.3)	127 (8.6)	4 (30.8)	100 (26.1)
Symptomatic	1,640 (87.7)	1,348 (91.4)	9 (69.2)	283 (73.9)
**Timing of maternal infection^¶^**	**[1,851]**	**[1,440]**	**[14]**	**[398]**
≤7 days before delivery	740 (40.0)	456 (31.7)	11 (84.6)	273 (68.6)
8–10 days before delivery	77 (4.2)	56 (3.9)	1 (7.7)	20 (5.0)
>10 days before delivery	1,034 (55.9)	928 (64.4)	1 (7.7)	105 (26.4)
Median (IQR) days from mother’s first positive test to delivery	17 (2–53)	28 (3–63)	1 (0–4)	2 (0–12)
Maximum days from mother’s first positive test to delivery	191	191	12	132
**Gestational age at birth**	**[2,692]**	**[2,085]**	**[16]**	**[591]**
Term (≥37 wks)	2,349 (87.3)	1,849 (88.7)	8 (50)	492 (83.2)
Late preterm (34–36 wks)	237 (8.8)	168 (8.1)	3 (18.8)	66 (11.2)
Moderate to very preterm (<34 wks)	106 (3.9)	68 (3.3)	5 (31.3)	33 (5.6)
Infant ICU admission of term infants** n/N (%)	**276/2,315 (11.9)**	**202/1,818 (11.1)**	**1/8 (12.5)**	**73/489 (14.9)**

**Abbreviations:** ICU = intensive care unit; IQR = interquartile range; RT-PCR = reverse transcription–polymerase chain reaction.

*Including California [excluding Los Angeles County], Houston, Los Angeles County, Michigan, Minnesota, Nebraska, New Jersey, North Dakota, Oklahoma, Pennsylvania [excluding Philadelphia], Puerto Rico, Tennessee, and Vermont.

^†^ A total of 313 (10.9%) live births were reported as not tested during birth hospitalization, the remainder had no testing results reported.

^§^ First positive test result occurred on the second day of life for 11 infants, on the third day for four, and on the fourth day for one.

^¶^ Defined as either date of specimen collection for first positive test or symptom onset if exact date of collection was missing.

**Among term (≥37 weeks) infants only. Reason for admission could include need for isolation of an otherwise asymptomatic infant based on possible SARS-CoV-2 exposure.

Eight of the infants with positive test results were born preterm (26–35 weeks); all were admitted to a neonatal ICU (NICU) without indications reported. Among the eight term infants with positive test results, one was admitted to a NICU for fever and receipt of supplemental oxygen, one had no information on NICU admission, and the remaining six were not admitted to a NICU. No neonatal immunoglobulin M or pregnancy-related specimen (e.g., placental tissue or amniotic fluid) testing was reported; thus, routes of transmission (in utero, peripartum, or postnatal) could not be assessed.

## Discussion

In this analysis of COVID-19 SET-NET data from 16 jurisdictions, the proportion of preterm live births among women with SARS-CoV-2 infection during pregnancy (12.9%) was higher than that in the general population in 2019 (10.2%) (*4*), suggesting that pregnant women with SARS-CoV-2 infection might be at risk for preterm delivery. These data are preliminary and describe primarily women with second and third trimester infection, and findings are subject to change pending completion of pregnancy for all women in the cohort and enhanced efforts to improve reporting of gestational age. This finding is consistent with other CDC reports describing higher proportions of preterm births among women hospitalized at the time of SARS-CoV-2 infection (*2*,*3*) and includes outcomes for women hospitalized as well as those not hospitalized at the time of infection (representing a population including persons with less severe illness). Increased frequency of preterm births was also described in a living, systematic review of SARS-CoV-2 infection in pregnancy (*5*). In contrast, a prospective cohort study of 253 infants found no difference in proportion of preterm birth or infant ICU admission between those born to women with positive SARS-CoV-2 test results and those born to women with suspected SARS-CoV-2 but negative test results (*6*), although the difference in findings between these two studies might be attributable to differences in case ascertainment, methodology, data collection, and sample size. Studies comparing pregnant women with and without COVID-19 are needed to assess the actual risk of preterm birth.

Non-Hispanic Black and Hispanic women were disproportionally represented in this surveillance cohort. Racial and ethnic disparities exist for maternal morbidity, mortality, and adverse birth outcomes (*7*–*9*), and the higher incidence and increased severity of COVID-19 among women of color might widen these disparities.*** Further surveillance efforts, including reporting by additional jurisdictions to improve representativeness, and careful analysis of outcomes by race and ethnicity, will permit more direct and targeted public health action.

Information regarding the frequency and severity of perinatal (potentially including in utero, peripartum, and postnatal) infection is lacking. The American Academy of Pediatrics and CDC recommend testing all infants born to mothers with suspected or confirmed COVID-19.^†††^ However, testing results were infrequently reported in this cohort. Perinatal infection was uncommon (2.6%) among infants known to have been tested for SARS-CoV-2 and occurred primarily among infants born to women with infection within 1 week of delivery. Among the infants with positive test results, one half were born preterm, which might reflect higher rates of screening in the ICU. These findings also support the growing evidence that although severe COVID-19 does occur in neonates the majority of term neonates experience asymptomatic infection or mild disease^§§§^; however, information on long term outcomes among exposed infants is unknown. 

The findings of this report are subject to at least six limitations. First, completeness of variables, particularly those ascertained through interviews or medical record abstraction, varied by jurisdiction. Statistical comparisons by maternal symptom status should be interpreted with caution given that symptom status was missing for a substantial proportion. Ongoing efforts to increase matching reported information with existing data sources has improved case ascertainment and completion of critical data elements. Testing and reporting might be more frequent among women with more severe illness or adverse birth outcomes. Second, these data are not nationally representative and include a higher frequency of Hispanic women compared with all women of reproductive age in national case surveillance data (*1*). Third, ascertainment of pregnancy loss depends on linkages to existing data sources (e.g., fetal death reporting), and potential underascertainment of this outcome limits comparison with national estimates. Fourth, few women with first trimester infection and completed pregnancy have been reported to date, limiting ability to evaluate adverse outcomes that might be more likely to be affected by infection earlier in pregnancy, such as birth defects. Fifth, risk factors (e.g., history of previous preterm birth) and clinical details associated with preterm delivery (e.g., spontaneous versus iatrogenic for maternal or fetal indications) were not assessed. Finally, a large proportion of infants had no testing reported. Positive SARS-CoV-2 RT-PCR results are reportable, and this percent positivity is likely an overestimate if negative testing was less often reported. Despite these limitations, this report describes a large population-based cohort with completed pregnancy outcomes and some infant testing.

These data can help to inform and counsel persons who acquire COVID-19 during pregnancy about potential risk to their pregnancy and infants; however, the risks associated with infection early in pregnancy and long-term infant outcomes remain unclear. SET-NET will continue to follow pregnancies affected by SARS-CoV-2 through completion of pregnancy and infants until age 6 months to guide clinical and public health practice. Longer-term investigation into solutions to alleviate underlying inequities in social determinants of health associated with disparities in maternal morbidity, mortality, and adverse pregnancy outcomes, and effectively addressing these inequities, could reduce the prevalence of conditions and experiences that might amplify risks from COVID-19. It is important that health care providers counsel pregnant women that SARS-CoV-2 infection might increase the risk for preterm birth and that infants born to women with infection identified >14 days before delivery might have a lower risk of having test results positive to SARS-CoV-2. Pregnant women and their household members should follow recommended infection prevention measures, including wearing a mask, social distancing, and frequent handwashing when going out or interacting with others. In addition, pregnant women should continue measures to ensure their general health including staying up to date with annual influenza vaccination and continuing prenatal care appointments.

**Summary**

**What is already known about this topic?**

Pregnant women with SARS-CoV-2 infection are at increased risk for severe illness compared with nonpregnant women. Adverse pregnancy outcomes such as preterm birth and pregnancy loss have been reported.

**What is added by this report?**

Among 3,912 infants with known gestational age born to women with SARS-CoV-2 infection, 12.9% were preterm (<37 weeks), higher than a national estimate of 10.2%. Among 610 (21.3%) infants with testing results, 2.6% had positive SARS-CoV-2 results, primarily those born to women with infection at delivery.

**What are the implications for public health practice?**

These findings can inform clinical practice, public health practice, and policy. It is important that providers counsel pregnant women on measures to prevent SARS-CoV-2 infection.

## Footnotes

* https://www.cdc.gov/coronavirus/2019-ncov/php/reporting-pui.html.

† https://www.cdc.gov/coronavirus/2019-ncov/need-extra-precautions/people-with-medical-conditions.html; https://www.cdc.gov/coronavirus/2019-ncov/if-you-are-sick/care-for-someone.html#face-covering.

§ https://assets.researchsquare.com/files/rs-90329/v1/e493691b-542c-47aa-a528-9e1dd9949879.pdf.

¶ https://wwwn.cdc.gov/nndss/conditions/coronavirus-disease-2019-covid-19/case-definition/2020/.

** California (excluding Los Angeles County), Georgia, Houston, Los Angeles County, Massachusetts, Michigan, Minnesota, Nebraska, New Jersey, New York (excluding New York City), North Dakota, Oklahoma, Pennsylvania (excluding Philadelphia), Puerto Rico, Tennessee, and Vermont.

†† https://www.cdc.gov/coronavirus/2019-ncov/cases-updates/special-populations/pregnancy-data-on-covid-19.html.

§§ 45 C.F.R. part 46, 21 C.F.R. part 56; 42 U.S.C. Sect. 241(d); 5 U.S.C. Sect. 552a; 44 U.S.C. Sect. 3501 et seq.

¶¶ Defined as weight <10th percentile for sex and gestational age using the INTERGROWTH-21st online percentile calculator. http://intergrowth21.ndog.ox.ac.uk.

*** https://www.cdc.gov/coronavirus/2019-ncov/covid-data/covidview/index.html.

††† https://services.aap.org/en/pages/2019-novel-coronavirus-covid-19-infections/clinical-guidance/faqs-management-of-infants-born-to-covid-19-mothers/; https://www.cdc.gov/coronavirus/2019-ncov/hcp/caring-for-newborns.html.

§§§ https://www.cdc.gov/coronavirus/2019-ncov/hcp/caring-for-newborns.html.
